# Prevalence of sarcopenia and its association with clinical outcomes in heart failure: An updated meta‐analysis and systematic review

**DOI:** 10.1002/clc.23970

**Published:** 2023-01-16

**Authors:** Ruzhao Chen, Jiachen Xu, Yuge Wang, Benyue Jiang, Xiao Xu, Yang Lan, Jiang Wang, Xiufang Lin

**Affiliations:** ^1^ The Center of Gerontology and Geriatrics/National Clinical Research Center for Geriatrics, West China Hospital Sichuan University Chengdu China; ^2^ Department of Medicine JingGangshan University Ji'an China

**Keywords:** heart failure, meta‐analysis, sarcopenia, systematic review, updated

## Abstract

**Background:**

Sarcopenia is thought to be strongly associated with heart failure, but meta‐analyses with sufficient samples are still lacking to accurately address its clinical situation.**Hypothesis**: Sarcopenia has a high prevalence in patients with heart failure and is closely related to adverse clinical outcomes.

**Methods:**

Relevant databases were systematically searched in October 2021 and updated in July 2022. The data with high heterogeneity were combined with random effects model.

**Results:**

Twenty‐one studies with 68,556 HF patients were included. The combined prevalence of sarcopenia in HF patients was 31%. Subgroup analysis found that the prevalence of sarcopenia in HF patients was 35% in Asia, 31% in Europe, 25% in the Americas, 31% in people aged ≥65 years, 25% in people with age <65 years, 28% in HF with reduced ejection fraction (HFrEF) patients and 18% in HF with preserved ejection fraction (HFpEF) patients. In addition, our analysis shows that sarcopenia in patients with HF is associated with an increased risk of poor prognosis, with a combined hazard ratio [HR] of 1.64 (95% confidence interval [CI] = 1.20–5.25), sarcopenia was also associated with poor outcomes in HFrEF patients with pooled HR of 2.77 (95% CI = 1.29–5.95). However, it was not associated with poor outcomes in HFpEF patients with pooled HR of 1.61 (95% CI = 0.82–3.16).

**Conclusions:**

The prevalence of sarcopenia is high in HF patients, and patients with HF, particularly those with reduced ejection fraction, are at high risk of adverse outcomes from sarcopenia. Therefore, early identification and intervention for sarcopenia were beneficial for improving the prognosis of HF patients.

**Abbreviations**
AHRQ: agency for healthcare research and quality
AKI: acute kidney injury
AWGS: Asian working group for sarcopenia
CHF: chronic heart failure
EWGSOP: European working group on sarcopenia in older people
HF: heart failure
HFpEF: heart failure with preserved ejection fraction
HFrEF: heart failure with reduced ejection fraction
HR: hazard risk
ICD‐9: international classification of diseases, ninth revision
NOS: Newcastle Ottawa scale

## 1 INTRODUCTION

Heart failure (HF) is a group of chronic syndromes characterized by tachypnea and fatigue.^1^, ^2^ Sarcopenia is a type of aging‐associated muscle loss and its prevalence and progression are significantly associated with concurrent risk factors.^3^ Recent clinical studies have shown that patients with HF often have decreased motor ability owing to decreased peripheral blood flow, which is the main causative factor for sarcopenia.^4^ In addition, sarcopenia is one of the main causes of poor prognosis in older patients with HF owing to poor physical performance.^5^ The proportion of older adults with multiple comorbidities has been steadily increasing in modern society. Furthermore, the prevalence of sarcopenia in older adults with chronic diseases has gradually increased, especially in intensive care units.^6^

Although several recent clinical trials and reviews have attempted to evaluate the clinical outcomes and prevalence of sarcopenia in patients with HF, most of these studies are limited by the number of investigations and test size and have not accurately identified its prevalence and clinical outcome. However, exploring the clinical outcomes and prevalence of sarcopenia in patients with HF remains an important proposition, and a meta‐analysis with large sample size is urgently needed. There are several causes of increased sarcopenia in patients with HF. In previous research that used the European Working Group on sarcopenia in older people (EWGSOP) to define sarcopenia, the incidence of sarcopenia in European participants aged 40–79 years was found to be 1.6%.^7^ In another study, the sarcopenia incidence rate was reported to be 3.6% in 85‐year‐old British men and women.^8^ However, the prevalence of sarcopenia based on the Asian Working Group for sarcopenia (AWGS) definition, was 3.4% in Chinese men and women.^9^ A few studies have reported “sex” as an important influencing factor.^10^ Furthermore, there is no definitive meta‐analysis study on the epidemiology of sarcopenia in patients with different ejection fraction phenotypes. Therefore, it is important to perform a stratified subgroup analysis to determine the prevalence of sarcopenia.

To date, only one meta‐analysis has systematically evaluated the prevalence of sarcopenia and its association with clinical outcomes in patients with HF.^11^ However, this meta‐analysis comprised a relatively small sample size, and could not accurately identify the prevalence of sarcopenia in the participants with HF or its association with the clinical outcome in patients with HF. A key conclusion of the study was that sex may not be a factor contributing to the difference in the prevalence of sarcopenia in the HF population, although another study showed that sarcopenia was more prevalent in women than in men.^6^

Given these inconsistencies, we performed an updated meta‐analysis and systematic review of various potential influencing factors to determine whether HF is an important factor affecting the prevalence of sarcopenia, and to investigate whether sarcopenia is associated with adverse clinical outcomes in HF. Our specific aim was to investigate the epidemiological characteristics of sarcopenia in the HF population and to explore its association with poor prognosis in HF by performing an updated meta‐analysis based on recently published data from large participant studies across multiple centers worldwide.

## 2 MATERIALS AND METHODS

Following the 2021MA,^11^ we conducted an updated meta‐analysis and systematic review. The 2021MA protocol was also optimized and updated to include more sample size.

### 2.1 Search strategy

We searched all records published from database inception to July 2022 using the following mesh words: “heart failure,” and “sarcopenia.” During the search, two researchers (Xiao Xu and Bunye Jiang) were assigned to independently complete the literature search and match the final search results to ensure that the results were correct.

### 2.2 Study selection

All articles we retrieved will be initially assessed by article titles and abstracts, during which we strictly follow the “back‐to‐back” principle. Two researchers (Ruzhao Chen and Bunye Jiang) conduct separate screening of each title abstract according to predetermined screening criteria and then download and read the full text for further confirmation, during which if there is disagreement, two dispatched researchers. A third researcher will discuss and decide until a consensus is reached.

### 2.3 Inclusion and exclusion criteria

We developed the following screening criteria for screening the literature: The study subjects were adults (>18 years of age) with HF participants of any sex or ethnicity; Definite studies on the diagnosis of HF and sarcopenia based on consensus^12^, ^13^, ^14^, ^15^; Original studies investigating the prevalence or clinical outcome of HF with sarcopenia; The study types include randomized controlled study, cross‐sectional study, case‐control study, or cohort study. Articles were excluded if prevalence could not be calculated, or no relevant clinical outcomes were reported. We also excluded publications without original data, such as letters, literature reviews and meta‐analyses, conference literature, basic research, and case reports. Animal experiments and non‐English literature were also excluded.

### 2.4 Data extraction

For each study finally included two researchers (Ruzhao Chen and Xiao Xu) independently extracted the publication year and location of the study, study design, sex characteristics and age of the cohort, sample size, examine participants, follow‐up time, type of HF, diagnostic guidelines for sarcopenia, the prevalence of sarcopenia, and clinical outcomes of HF using a standardized data extraction form. Following independent data extraction, two researchers cross‐checked all extracted data and resolved disagreements by consultation until a consensus was reached.

### 2.5 Assessment of risk of bias

Two researchers (Yuge Wang and Jiachen Xu) independently assessed methodological quality using the Newcastle‐Ottawa Scale (NOS) for 13 cohort studies included in examine.^16^ The scale is divided into three main modules: Selection, comparability, and outcome. The scale has a maximum score of 9 stars, and setting ≥7 points can be considered high‐quality studies. At the same time, the 8 included cross‐sectional studies were evaluated using 11 checklists recommended by the Agency for Health Care Quality and Research (AHRQ).^17^ The system was quantified, If the answer is “yes,” the score item is scored as “1“; if the answer is “no” or “unclear,” the score item is scored as “0,” with a full score of 11, and Article quality was evaluated with ≥8 as high quality.

### 2.6 Statistical analysis

Stata Version 17.0 (Stata Corp) and R 4.2.1 was used for data analyses. We used Cochran's Q statistic and *I* ^2^ statistic to examine heterogeneity between studies, *I* ^2^ ≥ 50% represents substantial heterogeneity in statistical treatment. Meanwhile, we used a two‐tailed test, and we considered the difference statistically significant when the *p*‐value was ≤.05. For the results of data combination, if the heterogeneity is significant, we choose the random effects model to combine to obtain more conservative and reliable results; Otherwise, we use the fixed effects model to merge, and the forest map illustrates the merge results. According to the prevalence of HF combined with sarcopenia, to find out the source of heterogeneity in the data and consider the utility of clinical guidance, we performed subgroup analyses and regression analyses according to region, HF type, age, Population Source (outpatients/inpatients), sex, study design, sarcopenia definition, and literature quality. We used Begg's test and Egger's test to evaluate potential publication bias precisely. The Trimmed filling method was used to correct the effect of publication bias on meta‐analysis for those parts with bias. Moreover, we used sensitivity analysis to evaluate the stability of the overall combined results.

## 3 RESULT

### 3.1 The result of the study selection

**Figure 1:**
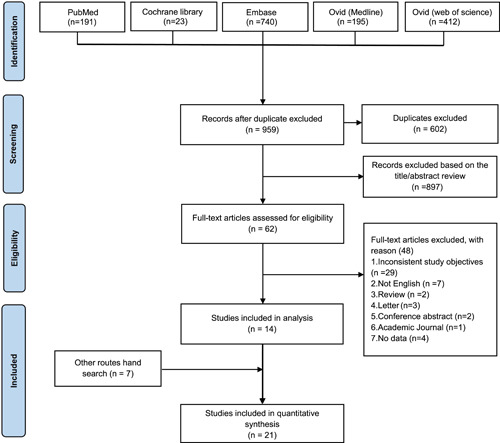
The flowchart for study screening

In addition to the studies included in 2021MA,^11^ a database search identified 10 additional published articles. Of the 1850 articles retrieved, duplicate and unrelated publications were excluded by screening the titles and abstracts of these articles based on predefined inclusion and exclusion criteria. Sixty‐two additional studies were reviewed further by reading the full text. Of these, 48 articles, including reviews, letters, conference abstracts, academic journals, and different study objectives, missing data, and non‐English literature were excluded as they did not meet the inclusion criteria. Based on tracked references and searches in the Google Scholar web search engine, four gray papers were included in the manual search. Thus, a total of 21 studies were included in this systematic review and meta‐analysis. Detailed information about the literature search and selection process is presented in a PRISMA flowchart in Figure 1.

### 3.2 Characteristics of included studies

Supporting Information: Table S1 summarized the detailed characteristics of the 21 observational studies, of which eight cross‐sectional and 13 cohort studies (10 prospective and three retrospective cohort studies). These studies were published between 2015 and 2022 and comprised 68,556 patients with HF that were included in this meta‐analysis and review. Of these 21 studies, 17 reported that the participants were from an outpatient or inpatient source. Twelve studies had clear diagnostic evidence of the ejection fraction phenotype in the HF population, eight of which reported reduced ejection fraction, two studies diagnosed preserved ejection fraction, and two others reported mixed reduced and preserved ejection fraction in the included population. The studies used a variety of criteria to define sarcopenia. Six studies used the AWGS criteria to define sarcopenia, whereas five used the definitions based on the EWGSOP.

### 3.3 Risk of bias in the included studies

Supporting Information: Table S2 provides the systemic quality evaluation using the NOS. The 13 cohort studies that were included in the analysis were of high quality with scores ranging from 6 to 8 and a total of 11 studies met the high‐quality setting. Supporting Information: Table S3 provides a point‐by‐point portrayal of the systemic quality evaluation of the eight included cross‐sectional that were included in the analysis using a checklist recommended by the AHRQ. The scores ranged from 4 to 8, with only one study showing high quality.

### 3.4 Epidemiology of sarcopenia in patients with HF

**Figure 2:**
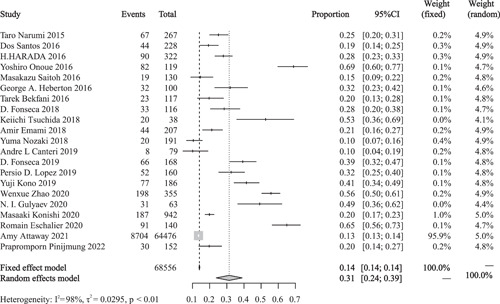
Prevalence of sarcopenia in patients with heart failure

Twenty‐one observational studies examined the epidemiology of sarcopenia in patients with HF. A random‐effects model was used to calculate the pooled effect size. As shown in Figure 2, the pooled prevalence of sarcopenia in patients with HF was 0.31 (95% confidence interval [CI] = 0.24–0.39, *p* < .01), and the studies showed significant heterogeneity (*I* ^2^ = 98%, *p* < .01).

### 3.5 Subgroup analyses and meta‐regressions

Based on the significant heterogeneity of our combined findings, subgroup and regression analyses of sarcopenia prevalence in patients with HF were further performed to explore the source of heterogeneity. It is important to note that the meta‐analysis of the prevalence of sarcopenia in HF was stratified based on study design, regions, HF type, age, sex, population source (outpatients/inpatients), sarcopenia definition, and study quality. In the subgroup analysis of the type of HF based on ejection fraction (Supporting Information: Figure S1), the meta‐analysis showed a higher prevalence (0.28; 95% CI = 0.17–0.38, *I* ^2^ = 96.0%, *p* < .01) of sarcopenia in patients with heart failure with reduced ejection fraction (HFrEF) compared with that (0.18; 95% CI = 0.15–0.22, *I* ^2^ = 0.0%, *p* < .01) in patients with heart failure with preserved ejection fraction (HFpEF). In the regional epidemiologic stratification study, the pooled prevalence reached 35% in the Asian population (95% CI = 0.22–0.48, *I* ^2^ = 98.0%, *p* < .01), 31% in the European population (95% CI = 0.17–0.45, *I* ^2^ = 96.0%, *p* < .01), and 25% in the American population (95% CI = 0.14–0.37, *I* ^2^ = 96.0%, *p* < .01) (Supporting Information: Figure S2). In the combined different age group study (Supporting Information: Figure S3), the meta‐analysis showed that the combined prevalence of HF with sarcopenia was significantly higher (0.31; 95% CI = 0.22–0.41, *I* ^2^ = 99.0%, *p* < .01) in participants ≥65 years of age compared with that in the <65 age group. A similar subgroup analysis in the population Source study showed that the prevalence of sarcopenia in the inpatient participants was almost twice that in the outpatient participants (Supporting Information: Figure S4). Furthermore, univariate meta‐regression analysis suggested that the origin of the participant (*p* = .03) may be one of the sources of heterogeneity (Table 1). No significant differences were found in other stratified studies or sources of heterogeneity (Supporting Information: Figures S5–S8, Table 2).

**Table 1:** Univariate meta‐regression of sarcopenia prevalence in heart failure

Variable	β (95% CI)	SE	*p*
Prevalence of sarcopenia in heart failure			
Study design: Retrospective cohort study versus others	0.15 (−0.07 to 0.38)	0.11	.17
Region: Asia versus others	0.07 (−0.10 to 0.23)	0.08	.40
Sex: Male versus female	0.02 (−0.15 to 0.19)	0.08	.78
Age: ≥65 years versus <65 years	1.07 (0.88 to 1.30)	0.01	.50
Population source: Inpatient versus outpatient	0.19 (0.02 to 0.37)	0.08	.03
Definition of sarcopenia: AWGS versus others	−0.08 (−0.26 to 0.10)	0.09	.37
Type of HF: HFrEF versus others	0.96 (0.75 to 1.23)	0.11	.73
High study quality: No versus yes	0.00 (−1.66 to 0.17)	0.08	.99
Poor clinical outcomes of sarcopenia in heart failure			
Mortality versus others	1.23 (0.50 to 3.04)	0.49	.62

Abbreviations: AWGS, Asian Working Group for Sarcopenia; HFrEF, Heart failure with reduced ejection fraction.

**Table 2:** Subgroup analyses of the prevalence of sarcopenia in heart failure patients

Category	*N*‐comparisons, participants	Prevalence	95% CI	*I* ^2^ (%)	*p* (z‐text)	Effect model
Total	*n* = 21, 68,556 participants	0.31	(0.24–0.39)	98.0	<.01	Random
Study design						
Cross‐sectional study	*n* = 8, 65,843 participants	0.29	(0.17–0.41)	98.0	<.01	Random
Prospective cohort study	*n* = 10, 2334 participants	0.29	(0.19–0.40)	95.0	<.01	Random
Retrospective cohort study	*n* = 3, 379 participants	0.44	(0.21–0.68)	96.0	<.01	Random
Regions						
Asia	*n* = 9, 2572 participants	0.35	(0.22–0.48)	98.0	<.01	Random
Europe	*n* = 7, 1001 participants	0.31	(0.17–0.45)	96.0	<.01	Random
Americas	*n* = 5, 64,983 participants	0.25	(0.14–0.37)	96.0	<.01	Random
Sex						
Male	*n* = 13, 29,541 participants	0.34	(0.23–0.45)	97.0	<.01	Random
Female	*n* = 13, 37,738 participants	0.32	(0.20–0.43)	96.0	<.01	Random
Age						
<65 years	*n* = 5, 65,012 participants	0.25	(0.13–0.36)	97.0	<.01	Random
≥65 years	*n* = 17, 68,020 participants	0.31	(0.22–0.41)	99.0	<.01	Random
Population source						
Outpatient	*n* = 6, 990 participants	0.20	(0.12–0.28)	90.0	<.01	Random
Inpatient	*n* = 11, 66,954 participants	0.39	(0.29–0.50)	99.0	<.01	Random
Definition of sarcopenia						
AWGS	*n* = 6, 2169 participants	0.26	(0.13–0.38)	97.0	<.01	Random
EWGSOP	*n* = 5, 830 participants	0.29	(0.11–0.48)	97.0	<.01	Random
Others	*n* = 10, 65,557 participants	0.36	(0.25–0.47)	98.0	<.01	Random
HF type						
HFrEF	*n* = 8, 1703 participants	0.28	(0.17–0.38)	96.0	<.01	Random
HFpEF	*n* = 2, 592 participants	0.18	(0.15–0.22)	0.0	<.01	Fixed
High study quality						
Yes	*n* = 12, 2881 participants	0.31	(0.21–0.42)	96.0	<.01	Random
No	*n* = 9, 65,675 participants	0.31	(0.20–0.43)	98.0	<.01	Random

Abbreviations: AWGS, Asian Working Group for Sarcopenia; EWGSOP, European Working Group on Sarcopenia in Older People; HFpEF, Heart failure with preserved ejection fraction; HFrEF, Heart failure with reduced ejection fraction.

### 3.6 Publication bias assessment

Results of the Begg's and Egger's tests showed a publication bias in the studies included in the analyses (*p* = .10 and <.01, respectively). The trim‐and‐fill method was used for evaluation and after two iterations of the linear method, the software estimated that the number of missing studies was zero and the results were stable.

### 3.7 Sensitivity analyses

We performed a sensitivity analysis to assess the robustness of the pooled HF with sarcopenia prevalence results by removing one study and pooling the rest. The sensitivity analyses showed no disruptive changes in the combined results, indicating that the combined results of the studies were robust (Supporting Information: Figure S9).

### 3.8 Prognostic effects of sarcopenia on patients with HF

**Figure 3:**
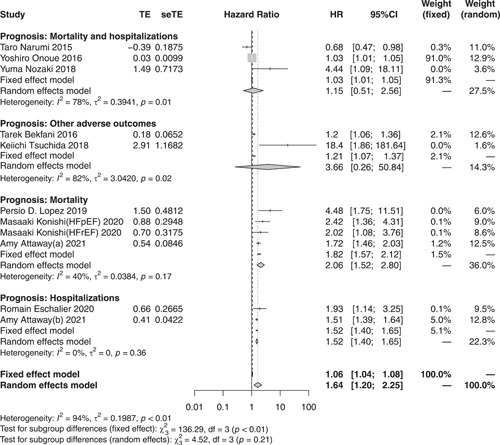
Subgroup analyses of clinical outcome

As shown in Figure 3, based on the random‐effects model, the pooled HR for poor prognosis in HF with sarcopenia was 1.64 (95% CI = 1.20–2.25, *I* ^2^ = 94.0%, *p* < .01), indicating that sarcopenia is associated with an increased risk of poor prognosis in HF. Subgroup analysis of adverse outcomes showed that sarcopenia was mainly associated with an increased risk of all‐cause mortality (HR: 2.06; 95% CI = 1.52–2.80, *I* ^2^ = 40.0%, *p* < .01) and hospitalization (HR: 1.52; 95% CI = 1.40–1.65, *I* ^2^ = 0.0%, *p* < .01) in patients with HF. Further subgroup analysis of the poor prognosis in the HF population with different ejection fractions showed that sarcopenia was associated with an increased risk of poor prognosis in patients with HF with different ejection fractions (HR: 2.01; 95% CI = 1.19–3.39, *I* ^2^ = 79.0%, *p* = .01), in those with HFpEF (HR: 1.61; 95% CI = 0.82–3.16, *I* ^2^ = 81.0%, *p* = .17), and in those with HFrEF (HR: 2.77; 95% CI = 1.29–5.95, *I* ^2^ = 48.0%, *p* = .01) (Supporting Information: Figure S10).

## 4 DISCUSSION

In this study, we examined the prevalence of sarcopenia in patients with HF and its association with adverse clinical outcomes. We found that the prevalence of sarcopenia was 31% in patients with HF. Some of our findings were consistent with MA results published in 2021.^11^ In addition, we determined the epidemiology of HF and sarcopenia by optimizing the meta‐analysis methods by including higher‐quality studies with large sample sizes and more refined statistical methods. Our findings suggest that cardiovascular physicians should focus more on screening for sarcopenia, particularly on stratified screening based on age and type of HF, and promptly apply effective prevention programs such as resistance training to lower the prevalence of sarcopenia and the risk of poor prognosis.^18^

According to a previous report, the prevalence of sarcopenia was 6%–12% in a large group of healthy participants.^19^ Our result showed that the combined prevalence of sarcopenia in patients with HF was close to 31%, which is consistent with the findings of 2021MA.^11^ We speculate that the high prevalence of sarcopenia in patients with HF may be attributed to the following reasons: peripheral blood reduction due to HF limits the patient's exercise capacity and thus directly or indirectly leads to a decrease in muscle mass.^4^ Second, HF‐induced chronic increase in vascular resistance leads to reduced skeletal muscle perfusion and hypoxia, which increases the accumulation of metabolites in the body and activates metabolic reflexes.^20^

Stratification of the studies by region revealed that the prevalence of sarcopenia was lower in European and American than in Asian countries. We speculate that this may be because most of the included Asian countries represent developing nations that may have lower nutritional levels compared to the developed countries. Malnutrition predisposes individuals to impaired muscle formation, which affects muscle mass and function.^21^ Another likely reason is the availability of higher levels of medical care and awareness of sarcopenia leading to early interventions in developed countries, whereas most hospitals and nursing homes in developing countries still have significant shortcomings in the understanding and control of sarcopenia.

The subgroup analysis showed that the prevalence of sarcopenia was higher in patients with HF with decreased ejection fraction than that in patients with preserved ejection fraction. In a prospective study conducted in European countries, the prevalence of sarcopenia with preserved ejection fraction was reported to be 19.7,^22^ which is consistent with our results. The results of the study by Kenisha et al. on sarcopenia in patients with different ejection fractions are also consistent with our findings.^23^ However, we believe that a simple comparison of the prevalence of sarcopenia in different HF phenotypes may be inappropriate owing to the influences of age and other diagnostic criteria for sarcopenia.^23^

Age subgroup analysis showed that sarcopenia was more prevalent in HF subjects aged ≥75 years than in those aged <75 years. The results of this study suggest that aging may play an important role in sarcopenia risk. According to Iannuzzi‐Sucich et al., the prevalence of sarcopenia increases with age.^24^ The decline in skeletal muscle mass, strength, and function with age seems undisputable although the exact reasons for the high proportion of sarcopenia in older adults remain unclear. Some studies point to a gradual decrease in muscle fibers after the age of 50,^25^ and another study suggests that aging‐induced hormone reduction may be an important contributing factor to muscle mass loss.^26^

Next, we performed subgroup analyses based on sex. Several epidemiological studies on sarcopenia in patients with HF have indicated a lower prevalence of sarcopenia in males than in females.^27^, ^28^, ^29^, ^30^ However, the results of the MA published in 2021 showed that the proportion of sarcopenia did not differ between the sexes.^11^ In the current study comprising 66,543 subjects, we found that the prevalence of sarcopenia was slightly higher (by three percentage points) in men than that in women in the HF group. The precise reason for this discrepancy remains unknown. We speculate that the bias of the selected and inconsistencies in the sarcopenia diagnostic scale may have contributed to this divergence. According to a previous report, sarcopenia is higher in men than in women owing to the reduction in the endogenous production of testosterone in older men and women, which results in sarcopenia and great muscle mass loss in men than in women.^31^

In a subgroup analysis based on population source, the prevalence of sarcopenia was found to be higher in inpatients with HF compared with that in outpatients, which is consistent with the results of 2021MA.^11^ This may be because patients diagnosed and treated in the outpatient clinic have mild symptoms and are likely to have early stages of HF with lower levels of HF‐associated sarcopenia.

Thus, the results of this study demonstrate that sarcopenia significantly increases the risk of adverse outcomes in patients with HF, including death and rehospitalization. In addition, it increases the risk of falls and hospitalization.^32^, ^33^ Multiple studies have shown that there may be common pathophysiological changes in sarcopenia and HF. Local or systemic factors, such as decreased physical activity, reduced body intake, malabsorption, systemic inflammation, and oxidative stress in patients with HF can cause muscle damage,^5^, ^34^, ^35^, ^36^ leading to adverse outcomes.^11^ We also found that patients with sarcopenia complicated with HFrEF had a higher risk of adverse outcomes, consistent with the fact that decreased left ventricular ejection fraction is associated with different degrees of muscular atrophy.^11^ Previous studies have shown that left ventricular ejection fraction predicts the occurrence of adverse outcomes and is very important in the management of patients with HF.^11^ In addition, sarcopenia impairs cardiopulmonary function in patients with HFrEF.^37^ Thus, although sarcopenia and HFrEF appear to be associated, the relationship between them remains unclear. Further studies are needed to explore the relationship between sarcopenia and HFrEF to reduce the occurrence of adverse outcomes in patients with HF complicated by sarcopenia, improve their prognosis and quality of life, and reduce the burden on their families and society.

### 4.1 Strengths and limitations

This study has several advantages and disadvantages. The greatest advantage of this paper is the optimization of the participants and research strategy based on original research results, and a better understanding of the epidemiology of sarcopenia in HF participants; Second, this is the first meta‐analysis for different HF ejection fraction phenotypes; Furthermore, we improve the exploration of study heterogeneity based on the univariate regression analysis method. Finally, although we have tried to strengthen the study protocol as much as possible, our results must be interpreted with caution owing to the following limitations. First, all the studies included in our meta‐analysis were observational studies, which may have influenced the results because of their subjective nature. Second, we limited the language of the published literature to English, which may have led to the omission of literature data in other languages, which may be a reason for publication bias in our study; Third, although most studies performed multivariate analysis to investigate the association between sarcopenia and poor prognosis in HF, not all studies adjusted for the same confounders, which may have led to under‐ or overestimated results. Finally, we could not investigate the prevalence of sarcopenia in people with HF of different sex backgrounds based on individual factors alone.

Thus, the complex association and mechanism between different HF phenotypes and sarcopenia appear worthy of further exploration. Further assessments of the association between sarcopenia and different HF phenotypes need to be supported by more original clinical studies with background correction of the participants.

## 5 CONCLUSIONS

The prevalence of sarcopenia is high in patients with HF and those with reduced ejection fraction have an increased risk of adverse outcomes. Therefore, early identification and interventions for sarcopenia may be beneficial in improving the prognosis of patients with HF.

## AUTHOR CONTRIBUTIONS

Ruzhao Chen designed the study. Jiachen Xu and Benyue Jiang contributed to the collection of literature, acquisition, analysis, and management of data. Yuge Wang drafted the manuscript. Xiao Xu, Yang Lan, Xiufang Lin, and Jiang Wang contributed to writing and proofreading the manuscript. All authors contributed to the manuscript for important intellectual content and approved the final submission of the manuscript.

## CONFLICT OF INTEREST

The authors declare no conflict of interest.

## Supporting information

Supplementary information.

Supplementary information.

Supplementary information.

Supplementary information.

Supplementary information.

Supplementary information.

Supplementary information.

Supplementary information.

Supplementary information.

Supplementary information.

Supplementary information.

Supplementary information.

Supplementary information.

## Data Availability

All data generated or analyzed during this study are included in this published article.
