# Supplementary material for: Prevalence of sarcopenia and its association with clinical outcomes in heart failure: An updated meta‐analysis and systematic review
Source: Clin Cardiol. 2023 Jan 16;46(3):260–8. doi: 10.1002/clc.23970 (PMC10018088; doi:10.1002/clc.23970)
Supplement: Supplementary file 3 — Supplementary information. [file CLC-46-260-s001.pdf]

Study	Events	Total	Proportion	95%CI	Weight (fixed)	Weight (random)
**Age: ≥65years**						
Taro Narumi 2015	67	267	0.25	[0.20; 0.31]	0.1%	4.6%
Dos Santos 2016	44	228	0.19	[0.14; 0.25]	0.1%	4.6%
H.HARADA 2016	90	322	0.28	[0.23; 0.33]	0.1%	4.6%
Masakazu Saitoh 2016	19	130	0.15	[0.09; 0.22]	0.0%	4.6%
Tarek Bekfani 2016	23	117	0.20	[0.13; 0.28]	0.0%	4.5%
Yoshiro Onoue 2016	82	119	0.69	[0.60; 0.77]	0.0%	4.5%
Amir Emami 2018	44	207	0.21	[0.16; 0.27]	0.1%	4.6%
Keiichi Tsuchida 2018	20	38	0.53	[0.36; 0.69]	0.0%	3.9%
Yuma Nozaki 2018	20	191	0.10	[0.07; 0.16]	0.1%	4.7%
Andre L Canteri 2019	8	79	0.10	[0.04; 0.19]	0.0%	4.6%
Persio D. Lopez 2019	52	160	0.32	[0.25; 0.40]	0.0%	4.5%
Yuji Kono 2019	77	186	0.41	[0.34; 0.49]	0.0%	4.6%
Masaaki Konishi 2020	187	942	0.20	[0.17; 0.23]	0.3%	4.7%
N. I. Gulyaev 2020	31	63	0.49	[0.36; 0.62]	0.0%	4.2%
Romain Eschalier 2020	91	140	0.65	[0.56; 0.73]	0.0%	4.5%
Wenxue Zhao 2020	198	355	0.56	[0.50; 0.61]	0.1%	4.6%
Amy Attaway(b) 2021	4695	64476	0.07	[0.07; 0.07]	45.6%	4.7%
Fixed effect model		68020	0.08	[0.07; 0.08]	46.6%	—
Random effects model			0.31	[0.22; 0.41]	—	77.2%
Heterogeneity: $I^2 = 99\%$, $\tau^2 = 0.0377$, $p < 0.01$						
**Age: <65years**						
George A. Heberton 2016	32	100	0.32	[0.23; 0.42]	0.0%	4.4%
D. Fonseca 2018	33	116	0.28	[0.20; 0.38]	0.0%	4.5%
D. Fonseca 2019	66	168	0.39	[0.32; 0.47]	0.0%	4.5%
Amy Attaway(a) 2021	3978	64476	0.06	[0.06; 0.06]	53.2%	4.7%
Prapromporn Pinijmung 2022	30	152	0.20	[0.14; 0.27]	0.0%	4.6%
Fixed effect model		65012	0.06	[0.06; 0.06]	53.4%	—
Random effects model			0.25	[0.13; 0.36]	—	22.8%
Heterogeneity: $I^2 = 97\%$, $\tau^2 = 0.0161$, $p < 0.01$						
**Fixed effect model**		**133032**	**0.07**	**[0.07; 0.07]**	**100.0%**	**—**
**Random effects model**			**0.30**	**[0.22; 0.38]**	**—**	**100.0%**

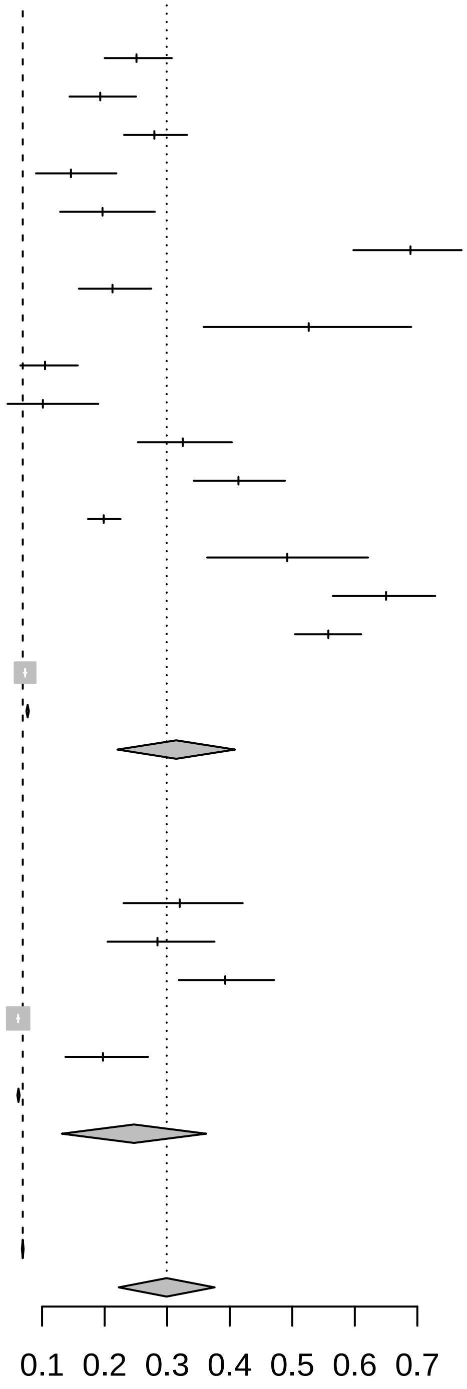

Heterogeneity: $I^2 = 99\%$, $\tau^2 = 0.0323$, $p < 0.01$
Test for subgroup differences (fixed effect): $\chi^2_1 = 111.34$, df = 1 ($p < 0.01$)
Test for subgroup differences (random effects): $\chi^2_1 = 0.79$, df = 1 ($p = 0.37$)

Supplementary Figure S3. Subgroup analysis for the prevalence of sarcopenia in the heart failure patients according to Age.
