# Supplementary material for: Prevalence of sarcopenia and its association with clinical outcomes in heart failure: An updated meta‐analysis and systematic review
Source: Clin Cardiol. 2023 Jan 16;46(3):260–8. doi: 10.1002/clc.23970 (PMC10018088; doi:10.1002/clc.23970)
Supplement: Supplementary file 4 — Supplementary information. [file CLC-46-260-s007.pdf]

Study	Events	Total	Proportion	95%CI	Weight (fixed)	Weight (random)
**Population Source: Inpatient**						
H.HARADA 2016	90	322	0.28	[0.23; 0.33]	0.3%	6.0%
Yoshiro Onoue 2016	82	119	0.69	[0.60; 0.77]	0.1%	5.8%
George A. Heberton 2016	32	100	0.32	[0.23; 0.42]	0.1%	5.7%
Keiichi Tsuchida 2018	20	38	0.53	[0.36; 0.69]	0.0%	5.1%
Da Fonseca 2018	33	116	0.28	[0.20; 0.38]	0.1%	5.8%
Persio D. Lopez 2019	52	160	0.32	[0.25; 0.40]	0.1%	5.9%
Yuji Kono 2019	77	186	0.41	[0.34; 0.49]	0.1%	5.9%
Masaaki Konishi 2020	187	942	0.20	[0.17; 0.23]	1.0%	6.1%
Romain Eschalier 2020	91	140	0.65	[0.56; 0.73]	0.1%	5.8%
Wenxue Zhao 2020	198	355	0.56	[0.50; 0.61]	0.3%	6.0%
Amy Attaway 2021	8704	64476	0.13	[0.13; 0.14]	96.5%	6.1%
Fixed effect model		66954	0.14	[0.14; 0.14]	98.8%	—
Random effects model			0.39	[0.29; 0.50]	—	64.3%
Heterogeneity: $I^2 = 99\%$, $\tau^2 = 0.0329$, $p < 0.01$						
**Population Source: Outpatient**						
Dos Santos 2016	44	228	0.19	[0.14; 0.25]	0.3%	6.0%
Tarek Bekfani 2016	23	117	0.20	[0.13; 0.28]	0.1%	5.9%
Amir Emami 2018	44	207	0.21	[0.16; 0.27]	0.2%	6.0%
Yuma Nozaki 2018	20	191	0.10	[0.07; 0.16]	0.4%	6.0%
Da Fonseca 2019	66	168	0.39	[0.32; 0.47]	0.1%	5.9%
Andre L Canteri 2019	8	79	0.10	[0.04; 0.19]	0.2%	5.9%
Fixed effect model		990	0.18	[0.16; 0.20]	1.2%	—
Random effects model			0.20	[0.12; 0.28]	—	35.7%
Heterogeneity: $I^2 = 90\%$, $\tau^2 = 0.0098$, $p < 0.01$						
**Fixed effect model**		**67944**	**0.14**	**[0.14; 0.14]**	**100.0%**	**—**
**Random effects model**			**0.33**	**[0.24; 0.41]**	**—**	**100.0%**

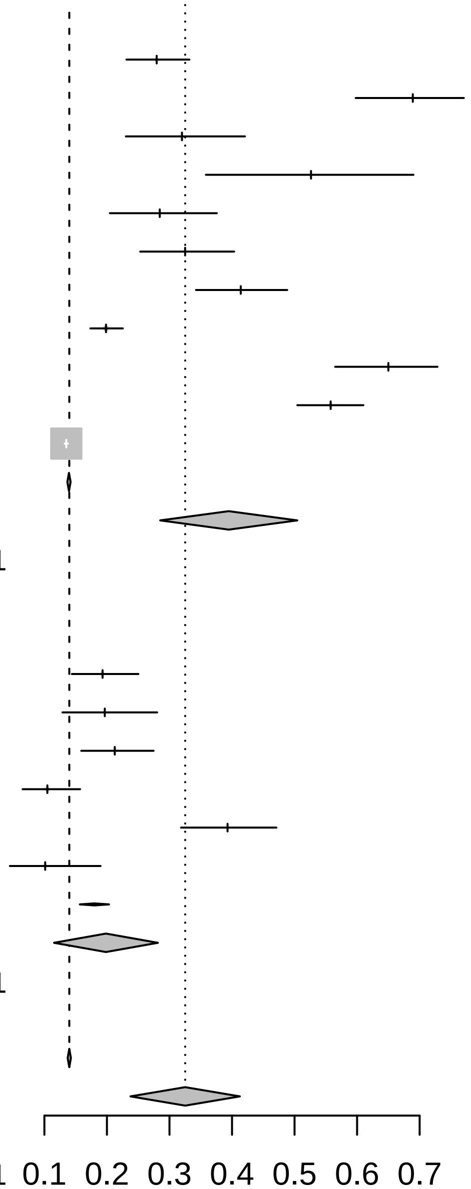

Heterogeneity: $I^2 = 98\%$, $\tau^2 = 0.0325$, $p < 0.01$

Test for subgroup differences (common effect): $\chi^2_1 = 11.53$, df = 1 ($p < 0.01$)

Test for subgroup differences (random effects): $\chi^2_1 = 7.83$, df = 1 ($p < 0.01$)

Supplementary Figure S4. Subgroup analysis for the prevalence of sarcopenia in the heart failure patients according to population source.
