# Supplementary material for: Prevalence of sarcopenia and its association with clinical outcomes in heart failure: An updated meta‐analysis and systematic review
Source: Clin Cardiol. 2023 Jan 16;46(3):260–8. doi: 10.1002/clc.23970 (PMC10018088; doi:10.1002/clc.23970)
Supplement: Supplementary file 5 — Supplementary information. [file CLC-46-260-s009.pdf]

Study	Events	Total	Proportion	95%CI	Weight (fixed)	Weight (random)
**Sex: Female**						
Dos Santos 2016	10	47	0.21	[0.11; 0.36]	0.0%	3.8%
George A. Heberton 2016	7	23	0.30	[0.13; 0.53]	0.0%	3.4%
H.HARADA 2016	56	135	0.41	[0.33; 0.50]	0.1%	3.9%
Masakazu Saitoh 2016	1	25	0.04	[0.00; 0.20]	0.1%	4.0%
Tarek Bekfani 2016	3	38	0.08	[0.02; 0.21]	0.1%	3.9%
Yoshiro Onoue 2016	29	46	0.63	[0.48; 0.77]	0.0%	3.7%
Keiichi Tsuchida 2018	4	13	0.31	[0.09; 0.61]	0.0%	2.9%
Persio D. Lopez 2019	16	49	0.33	[0.20; 0.48]	0.0%	3.7%
Masaaki Konishi 2020	55	392	0.14	[0.11; 0.18]	0.6%	4.1%
Romain Eschalier 2020	37	58	0.64	[0.50; 0.76]	0.0%	3.8%
Wenxue Zhao 2020	93	148	0.63	[0.55; 0.71]	0.1%	4.0%
Amy Attaway 2021	4920	36719	0.13	[0.13; 0.14]	55.4%	4.1%
Prapromporn Pinijmung 2022	14	45	0.31	[0.18; 0.47]	0.0%	3.7%
Fixed effect model		37738	0.14	[0.13; 0.14]	56.6%	—
Random effects model			0.32	[0.20; 0.43]	—	48.9%
Heterogeneity: $I^2 = 96\%$, $\tau^2 = 0.0419$, $p < 0.01$						
**Sex: Male**						
Dos Santos 2016	36	181	0.20	[0.14; 0.26]	0.2%	4.0%
George A. Heberton 2016	25	77	0.32	[0.22; 0.44]	0.1%	3.9%
H.HARADA 2016	34	187	0.18	[0.13; 0.24]	0.2%	4.0%
Masakazu Saitoh 2016	18	105	0.17	[0.10; 0.26]	0.1%	4.0%
Tarek Bekfani 2016	20	79	0.25	[0.16; 0.36]	0.1%	3.9%
Yoshiro Onoue 2016	53	73	0.73	[0.61; 0.82]	0.1%	3.9%
Keiichi Tsuchida 2018	16	25	0.64	[0.43; 0.82]	0.0%	3.4%
Persio D. Lopez 2019	36	111	0.32	[0.24; 0.42]	0.1%	3.9%
Masaaki Konishi 2020	132	550	0.24	[0.20; 0.28]	0.5%	4.1%
Romain Eschalier 2020	54	82	0.66	[0.55; 0.76]	0.1%	3.9%
Wenxue Zhao 2020	105	207	0.51	[0.44; 0.58]	0.1%	4.0%
Amy Attaway 2021	3747	27757	0.13	[0.13; 0.14]	41.6%	4.1%
Prapromporn Pinijmung 2022	15	107	0.14	[0.08; 0.22]	0.2%	4.0%
Fixed effect model		29541	0.14	[0.14; 0.14]	43.4%	—
Random effects model			0.34	[0.23; 0.45]	—	51.1%
Heterogeneity: $I^2 = 97\%$, $\tau^2 = 0.0414$, $p < 0.01$						
**Fixed effect model**		**67279**	**0.14**	**[0.14; 0.14]**	**100.0%**	**—**
**Random effects model**			**0.33**	**[0.25; 0.41]**	**—**	**100.0%**

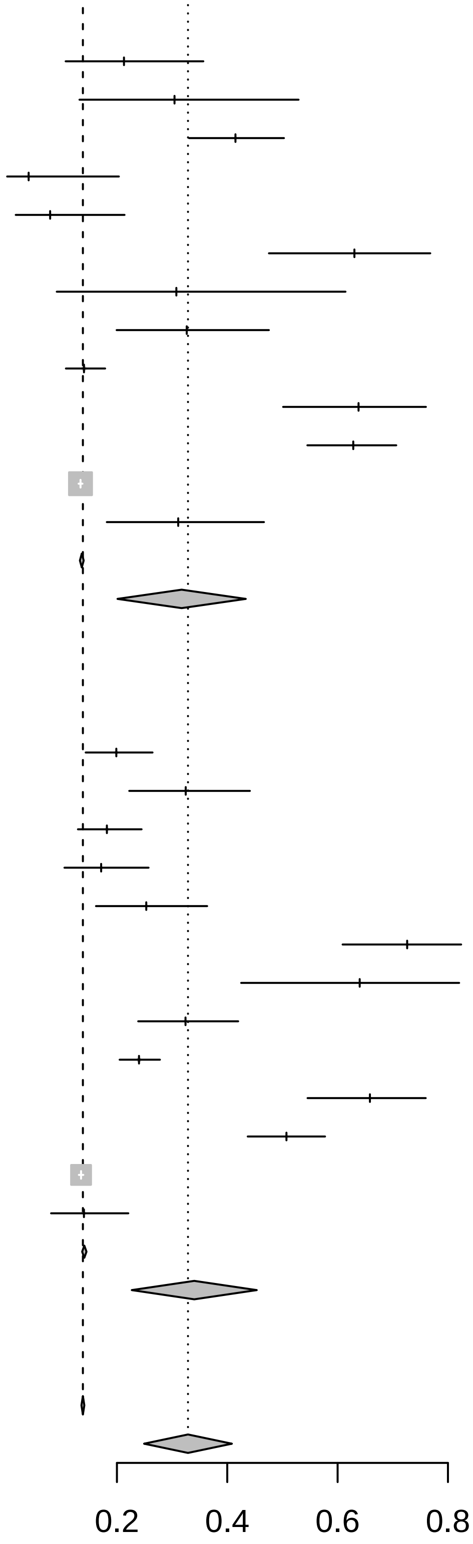

Heterogeneity: $I^2 = 97\%$, $\tau^2 = 0.0400$, $p < 0.01$
Test for subgroup differences (common effect): $\chi^2_1 = 2.93$, df = 1 ($p = 0.09$)
Test for subgroup differences (random effects): $\chi^2_1 = 0.08$, df = 1 ($p = 0.78$)

Supplementary Figure S5. Subgroup analysis for the prevalence of sarcopenia in the heart failure patients according to sex.
