# Supplementary material for: Prevalence of sarcopenia and its association with clinical outcomes in heart failure: An updated meta‐analysis and systematic review
Source: Clin Cardiol. 2023 Jan 16;46(3):260–8. doi: 10.1002/clc.23970 (PMC10018088; doi:10.1002/clc.23970)
Supplement: Supplementary file 6 — Supplementary information. [file CLC-46-260-s013.pdf]

Study	Events	Total	Proportion	95%CI	Weight (fixed)	Weight (random)
**Study desgin: Cross sectional study**						
Wenxue Zhao 2020	198	355	0.56	[0.50; 0.61]	0.2%	4.9%
Prapromporn Pinijmung 2022	30	152	0.20	[0.14; 0.27]	0.2%	4.8%
Dos Santos 2016	44	228	0.19	[0.14; 0.25]	0.3%	4.9%
Andre L Canteri 2019	8	79	0.10	[0.04; 0.19]	0.2%	4.8%
Amy Attaway 2021	8704	64476	0.13	[0.13; 0.14]	95.9%	5.0%
D. Fonseca 2019	66	168	0.39	[0.32; 0.47]	0.1%	4.8%
N. I. Gulyaev 2020	31	63	0.49	[0.36; 0.62]	0.0%	4.4%
H.HARADA 2016	90	322	0.28	[0.23; 0.33]	0.3%	4.9%
Fixed effect model		65843	0.14	[0.13; 0.14]	97.2%	—
Random effects model			0.29	[0.17; 0.41]	—	38.4%
Heterogeneity: $I^2 = 98\%$, $\tau^2 = 0.0272$, $p < 0.01$						
**Study desgin: Prospective cohort study**						
D. Fonseca 2018	33	116	0.28	[0.20; 0.38]	0.1%	4.7%
Keiichi Tsuchida 2018	20	38	0.53	[0.36; 0.69]	0.0%	4.1%
Amir Emami 2018	44	207	0.21	[0.16; 0.27]	0.2%	4.9%
Yuma Nozaki 2018	20	191	0.10	[0.07; 0.16]	0.4%	4.9%
Masaaki Konishi 2020	187	942	0.20	[0.17; 0.23]	1.0%	5.0%
Taro Narumi 2015	67	267	0.25	[0.20; 0.31]	0.2%	4.9%
Romain Eschalier 2020	91	140	0.65	[0.56; 0.73]	0.1%	4.7%
Masakazu Saitoh 2016	19	130	0.15	[0.09; 0.22]	0.2%	4.8%
Tarek Bekfani 2016	23	117	0.20	[0.13; 0.28]	0.1%	4.8%
Yuji Kono 2019	77	186	0.41	[0.34; 0.49]	0.1%	4.8%
Fixed effect model		2334	0.23	[0.21; 0.24]	2.5%	—
Random effects model			0.29	[0.19; 0.40]	—	47.5%
Heterogeneity: $I^2 = 95\%$, $\tau^2 = 0.0284$, $p < 0.01$						
**Study desgin: Retrospective cohort study**						
Yoshiro Onoue 2016	82	119	0.69	[0.60; 0.77]	0.1%	4.7%
George A. Heberton 2016	32	100	0.32	[0.23; 0.42]	0.1%	4.6%
Persio D. Lopez 2019	52	160	0.32	[0.25; 0.40]	0.1%	4.8%
Fixed effect model		379	0.44	[0.39; 0.49]	0.3%	—
Random effects model			0.44	[0.21; 0.68]	—	14.1%
Heterogeneity: $I^2 = 96\%$, $\tau^2 = 0.0430$, $p < 0.01$						
**Fixed effect model**		**68556**	**0.14**	**[0.14; 0.14]**	**100.0%**	**—**
**Random effects model**			**0.31**	**[0.24; 0.39]**	**—**	**100.0%**

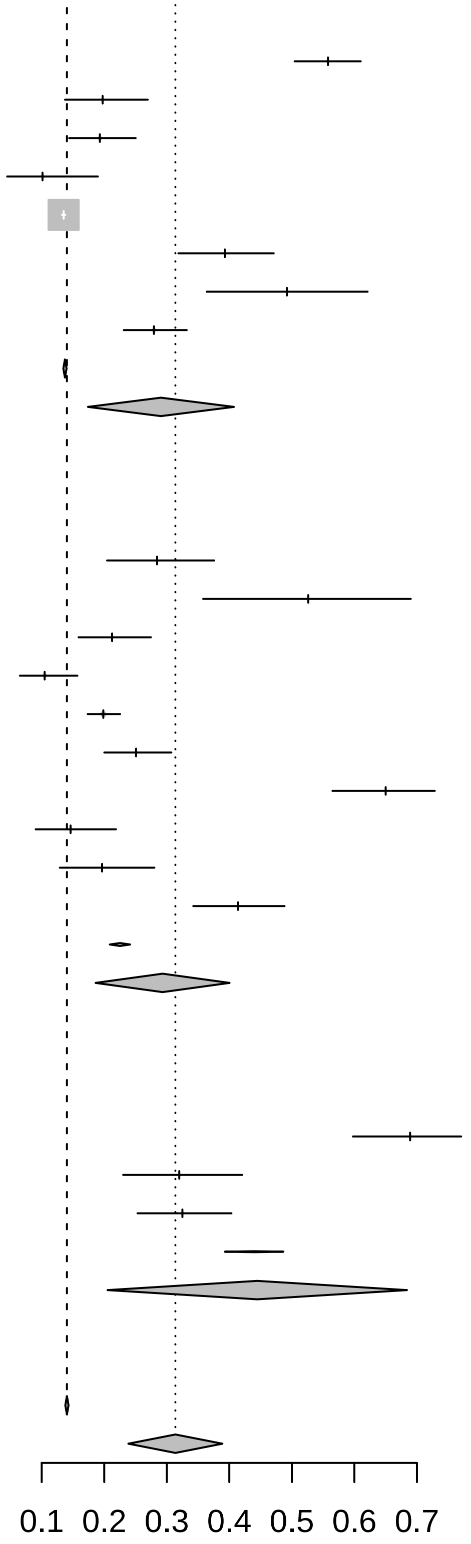

Heterogeneity: $I^2 = 98\%$, $\tau^2 = 0.0295$, $p < 0.01$

Test for subgroup differences (common effect): $\chi^2_2 = 266.17$, df = 2 ($p < 0.01$)

Test for subgroup differences (random effects): $\chi^2_2 = 1.41$, df = 2 ($p = 0.49$)

Supplementary Figure S6. Subgroup analysis for the prevalence of sarcopenia in the heart failure patients according to study design.
