# Supplementary material for: Prevalence of sarcopenia and its association with clinical outcomes in heart failure: An updated meta‐analysis and systematic review
Source: Clin Cardiol. 2023 Jan 16;46(3):260–8. doi: 10.1002/clc.23970 (PMC10018088; doi:10.1002/clc.23970)
Supplement: Supplementary file 7 — Supplementary information. [file CLC-46-260-s012.pdf]

| Study | Events | Total | Proportion | 95%CI | Weight (fixed) | Weight (random) |
|---|---|---|---|---|---|---|
| **Definition of Sarcopenia: EWGSOP** | | | | | | |
| Taro Narumi 2015 | 67 | 267 | 0.25 | [0.20; 0.31] | 0.2% | 4.9% |
| Dos Santos 2016 | 44 | 228 | 0.19 | [0.14; 0.25] | 0.3% | 4.9% |
| D. Fonseca 2018 | 33 | 116 | 0.28 | [0.20; 0.38] | 0.1% | 4.7% |
| Andre L Canteri 2019 | 8 | 79 | 0.10 | [0.04; 0.19] | 0.2% | 4.8% |
| Romain Eschalier 2020 | 91 | 140 | 0.65 | [0.56; 0.73] | 0.1% | 4.7% |
| Fixed effect model | | 830 | 0.26 | [0.23; 0.29] | 0.9% | — |
| Random effects model | | | 0.29 | [0.11; 0.48] | — | 24.0% |
| Heterogeneity: $I^2 = 97\%$, $\tau^2 = 0.0423$, $p < 0.01$ | | | | | | |
| **Definition of Sarcopenia: AWGS** | | | | | | |
| H.HARADA 2016 | 90 | 322 | 0.28 | [0.23; 0.33] | 0.3% | 4.9% |
| Amir Emami 2018 | 44 | 207 | 0.21 | [0.16; 0.27] | 0.2% | 4.9% |
| Yuma Nozaki 2018 | 20 | 191 | 0.10 | [0.07; 0.16] | 0.4% | 4.9% |
| Wenxue Zhao 2020 | 198 | 355 | 0.56 | [0.50; 0.61] | 0.2% | 4.9% |
| Masaaki Konishi 2020 | 187 | 942 | 0.20 | [0.17; 0.23] | 1.0% | 5.0% |
| Prapromporn Pinijmung 2022 | 30 | 152 | 0.20 | [0.14; 0.27] | 0.2% | 4.8% |
| Fixed effect model | | 2169 | 0.23 | [0.22; 0.25] | 2.3% | — |
| Random effects model | | | 0.26 | [0.13; 0.38] | — | 29.3% |
| Heterogeneity: $I^2 = 97\%$, $\tau^2 = 0.0240$, $p < 0.01$ | | | | | | |
| **Definition of Sarcopenia: Others** | | | | | | |
| Yoshiro Onoue 2016 | 82 | 119 | 0.69 | [0.60; 0.77] | 0.1% | 4.7% |
| Masakazu Saitoh 2016 | 19 | 130 | 0.15 | [0.09; 0.22] | 0.2% | 4.8% |
| George A. Heberton 2016 | 32 | 100 | 0.32 | [0.23; 0.42] | 0.1% | 4.6% |
| Tarek Bekfani 2016 | 23 | 117 | 0.20 | [0.13; 0.28] | 0.1% | 4.8% |
| Keiichi Tsuchida 2018 | 20 | 38 | 0.53 | [0.36; 0.69] | 0.0% | 4.1% |
| D. Fonseca 2019 | 66 | 168 | 0.39 | [0.32; 0.47] | 0.1% | 4.8% |
| Persio D. Lopez 2019 | 52 | 160 | 0.32 | [0.25; 0.40] | 0.1% | 4.8% |
| Yuji Kono 2019 | 77 | 186 | 0.41 | [0.34; 0.49] | 0.1% | 4.8% |
| N. I. Gulyaev 2020 | 31 | 63 | 0.49 | [0.36; 0.62] | 0.0% | 4.4% |
| Amy Attaway 2021 | 8704 | 64476 | 0.13 | [0.13; 0.14] | 95.9% | 5.0% |
| Fixed effect model | | 65557 | 0.14 | [0.13; 0.14] | 96.9% | — |
| Random effects model | | | 0.36 | [0.25; 0.47] | — | 46.7% |
| Heterogeneity: $I^2 = 98\%$, $\tau^2 = 0.0300$, $p < 0.01$ | | | | | | |
| **Fixed effect model** | | **68556** | **0.14** | **[0.14; 0.14]** | **100.0%** | **—** |
| **Random effects model** | | | **0.31** | **[0.24; 0.39]** | **—** | **100.0%** |

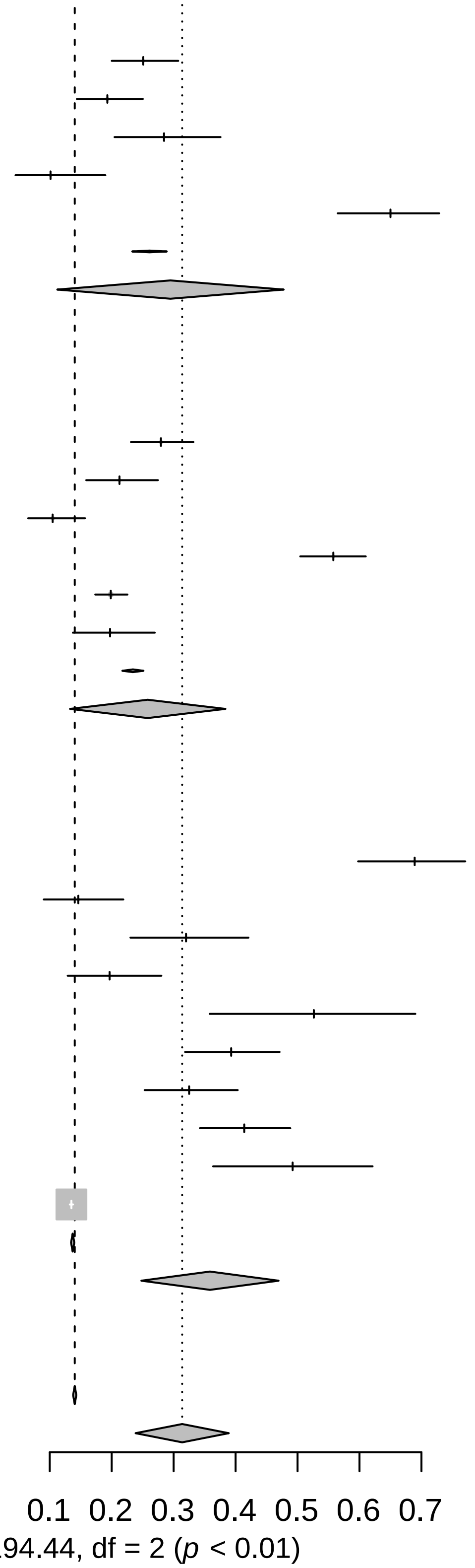


Heterogeneity: $I^2 = 98\%$, $\tau^2 = 0.0295$, $p < 0.01$

Test for subgroup differences (fixed effect): $\chi^2_2 = 194.44$, df = 2 ($p < 0.01$)

Test for subgroup differences (random effects): $\chi^2_2 = 1.42$, df = 2 ($p = 0.49$)

Supplementary Figure S7. Subgroup analysis for the prevalence of sarcopenia in the heart failure patients according to sarcopenia definition.
