# Supplementary material for: Prevalence of sarcopenia and its association with clinical outcomes in heart failure: An updated meta‐analysis and systematic review
Source: Clin Cardiol. 2023 Jan 16;46(3):260–8. doi: 10.1002/clc.23970 (PMC10018088; doi:10.1002/clc.23970)
Supplement: Supplementary file 11 — Supplementary information. [file CLC-46-260-s006.docx]

| **Supplementary table 1.the characteristics of included studies.** | | | | | | | | | |
| --- | --- | --- | --- | --- | --- | --- | --- | --- | --- |
| **Authors**  **(year)** | **Region** | **Study design** | **Sample number** | **Mean age(SD)** | **Participants** | **Type of HF** | **Definition of Sarcopenia** | **Rate%** | **Literature quality** |
| D. Fonseca 2019 | Brazil | Cross-sectional study | 168 | 57.0(8.2) | Outpatient patients from hospital | HFrEF | Others | 0.393 | 6 |
| H. HARADA 2016 | Japan | Cross-sectional study | 322 | 72.0(12) | Inpatient patients from from Institute of Health and Sports Sciences, Kurume University | CHF | AWGS | 0.280 | 8 |
| Andre L Canteri 2019 | Brazil | Cross-sectional study | 79 | 65.6(13) | Outpatient patients from database of  the Echocardiography Service | HFrEF | EWGSOP | 0.101 | 7 |
| Amy Attaway 2021 | USA | Cross-sectional study | 64,476 | _ | Inpatient from The Nationwide Inpatient Sample (NIS) | HF | ICD-9 | 0.135 | 4 |
| Masakazu Santos 2016 | Germany | Cross-sectional study | 228 | 68.8(9.6) | Outpatient patients from Charité Medical School, Campus Virchow-Klinikum, Berlin, Germany | HFrEF | EWGSOP | 0.195 | 7 |
| N. I. Gulyaev 2020 | Russia | Cross-sectional study | 63 | 77.2(7.7) | Patients from Hospital | CHF | Others | 0.492 | 5 |
| Prapromporn Pinijmung 2022 | Thailand | Cross-sectional study | 152 | 58.5(11.8) | Inpatient and Outpatient patients from Hospital | HF | AWGS | 0.198 | 6 |
| Taro Narumi 2015 | Japan | Prospective cohort study | 267 | 71.0(12) | Patients fromYamagata University Hospital | CFC | EWGSOP | 0.250 | 8 |
| Masaaki Konishi 2020 | Japan | Prospective cohort study | 942 | 79.0(8) | Inpatient Patients from Japan（Yokohama City University Medical Center） | HF | AWGS | 0.199 | 7 |
| Romain Eschalier 2020 | France | Prospective cohort study | 140 | 75.8(3.9) | Inpatient patients from Clermont-Ferrand University Hospital or the Durtol Cardiac Rehabilitation Center | ADHF | EWGSOP | 0.650 | 7 |
| Persio D. Lopez 2019 | USA | Retrospective cohort study | 160 | 66.3(13.8) | Inpatient patients from single urban community hospital. | HFrEF | Others | 0.325 | 7 |
| Yoshiro Onoue 2016 | Japan | Retrospective cohort study | 119 | 76.1(6.2) | Inpatient patients from Kumamoto University Hospital | HFrEF, HFpEF | Others | 0.689 | 7 |
| Masakazu Saitoh 2016 | Germany | Prospective observational study | 130 | 66.3(11.5) | Patients from Charité Medical School, Campus Virchow-Klinikum, Berlin, Germany | HFrEF | Others | 0.146 | 7 |
| Amir Emami 2018 | Germany | Prospective observational study | 207 | 67.3(10.1) | Outpatient patients from Department of Cardiology, Charité Medical School, Campus Virchow-Klinikum, Berlin, Germany | HFrEF, HFpEF | AWGS | 0.213 | 8 |
| Keiichi Tsuchida 2018 | Japan | Prospective observational study | 38 | 75.0(11.4) | Inpatient patients from Department of Cardiology, Niigata City General Hospital, Niigata, Japan | ADHF | Others | 0.526 | 6 |
| George A. Heberton 2016 | USA | Retrospective cohort study | 100 | 54.3(14.06) | Inpatient patients from Hospital | HF | Others | 0.320 | 7 |
| Tarek Bekfani 2016 | Germany | Prospective observational study | 117 | 69.8(8.5) | Outpatient patients from hospital | HFpEF | Others | 0.197 | 8 |
| Yuji Kono 2019 | Japan | Cross-sectional study | 186 | 79.8(9.64) | Inpatient from Fujita Health University Banbuntane Hotokukai Hospital | ADHF | Others | 0.413 | 8 |
| Wenxue Zhao2020 | China | Cross-sectional study | 355 | 71.01（9.41） | Inpatient patients from Hospital | HFrEF | AWGS | 0.558 | 5 |
| Yuma Nozaki 2018 | Japan | Prospective cohort study | 191 | 73.3(7.3) | Outpatient patients from hospital | HF | AWGS | 0.105 | 8 |
| Da Fonseca 2018 | Germany | Prospective cohort study | 116 | 55(9) | Inpatient patients from Hospital | HFrEF | European Working Group | 0.284 | 6 |

**HFrEF:** Heart failure with reduced ejection fraction; **HFpEF:**Heart failure with preserved ejection fraction ;**CHF:** chronic heart failure ; **CFC:** Cardiac failure congestive; **ADHF:** Acute Decompensated Heart Failure; **AWGS:** [Asian Working Group for Sarcopenia](https://pubmed.ncbi.nlm.nih.gov/32033882/); **EWGSOP:**European Working Group on Sarcopenia in Older People; **ICD-9:**International Classification of Diseases, Ninth Revision
