# Supplementary material for: Prevalence of sarcopenia and its association with clinical outcomes in heart failure: An updated meta‐analysis and systematic review
Source: Clin Cardiol. 2023 Jan 16;46(3):260–8. doi: 10.1002/clc.23970 (PMC10018088; doi:10.1002/clc.23970)
Supplement: Supplementary file 12 — Supplementary information. [file CLC-46-260-s004.doc]

| **Supplementary table 2.the NOS score for cohort studies.** | | | | | | | | | |
| --- | --- | --- | --- | --- | --- | --- | --- | --- | --- |
| **Study** | **Selection (1)** | | | | **Comparability (2)** | **Outcome (3)** | | | **Score** |
|
|  | Representativeness of the exposed cohort | Selection of the non-exposed cohort | Ascertainment of exposure | Demonstration that outcome of Interest was not Present at start of study | Comparability of cohorts on the basis of the design or analysis | Assessment of outcome | Was follow-up long enough for outcomes to occur | Adequacy of follow up of cohorts |  |
| George A. Heberton 2016 |  |  | ✮ | ✮ | ✮✮ | ✮ | ✮ | ✮ | 7 |
| Tarek Bekfani 2016 | ✮ | ✮ | ✮ | ✮ | ✮✮ | ✮ | ✮ |  | 8 |
| Masaaki Konishi 2020 |  |  | ✮ | ✮ | ✮✮ | ✮ | ✮ | ✮ | 7 |
| Romain Eschalier 2021 |  |  | ✮ | ✮ | ✮✮ | ✮ | ✮ | ✮ | 7 |
| Yuji Kono 2019 | ✮ | ✮ | ✮ | ✮ | ✮✮ | ✮ | ✮ |  | 8 |
| Persio D. Lopez 2019 |  |  | ✮ | ✮ | ✮✮ | ✮ | ✮ | ✮ | 7 |
| Keiichi Tsuchida 2018 | ✮ | ✮ | ✮ |  | ✮✮ | ✮ |  |  | 6 |
| Yoshiro Onoue 2016 |  |  | ✮ | ✮ | ✮✮ | ✮ | ✮ | ✮ | 7 |
| Taro Narumi 2015 | ✮ | ✮ | ✮ | ✮ | ✮✮ | ✮ |  | ✮ | 8 |
| Amir Emami 2018 | ✮ | ✮ | ✮ |  | ✮✮ | ✮ | ✮ | ✮ | 8 |
| Masakazu Saitoh 2016 |  |  | ✮ | ✮ | ✮✮ | ✮ | ✮ | ✮ | 7 |
| Yuma Nozaki 2018 | ✮ | ✮ | ✮ | ✮ | ✮ | ✮ | ✮ | ✮ | 8 |
| Da Fonseca 2018 | ✮ | ✮ | ✮ |  |  | ✮ | ✮ | ✮ | 6 |
