# Supplementary material for: Prevalence of sarcopenia and its association with clinical outcomes in heart failure: An updated meta‐analysis and systematic review
Source: Clin Cardiol. 2023 Jan 16;46(3):260–8. doi: 10.1002/clc.23970 (PMC10018088; doi:10.1002/clc.23970)
Supplement: Supplementary file 13 — Supplementary information. [file CLC-46-260-s003.docx]

| **Supplementary table 3.the AHRQ score for cross-sectional studies.** | | | | | | | | | | | | |
| --- | --- | --- | --- | --- | --- | --- | --- | --- | --- | --- | --- | --- |
| Study | (1)Define the source of information (survey , record review) | (2)List inclusion and exclusion criteria for exposed and unexposed subjects (cases and controls) or refer to previous publications | (3)Indicate time period used for identifying patients | (4)Indicate whether or not subjects were consecutive if not population-based | (5)Indicate if evaluators of subjective components of study were masked to other aspects of the status participants | (6)Describe any assessments undertaken for quality assurance purposes(e.g., test/retest of primary outcome measurements) | (7)Explain any patient exclusions from analysis | (8)Describe how confounding was assessed and/or controlled. | (9)If applicable, explain how missing data were handled in the analysis | (10)Summarize patient response rates and completeness of data collection | (11)Clarify what follow-up, if any, was expected and the percentage of patients for which incomplete data or follow-up was obtained | Score |
| D. Fonseca 2019 | 1 | 1 | 1 | 1 | 0 | 1 | 0 | 0 | 0 | 1 | 0 | 6 |
| H.HARADA 2016 | 1 | 1 | 1 | 1 | 0 | 1 | 1 | 1 | 0 | 1 | 0 | 8 |
| Andre L Canteri 2019 | 1 | 1 | 0 | 1 | 0 | 1 | 1 | 1 | 0 | 1 | 0 | 7 |
| Amy Attaway 2021 | 1 | 1 | 1 | 0 | 0 | 0 | 0 | 1 | 0 | 0 | 0 | 4 |
| Marcelo R. dos Santos 2016 | 1 | 1 | 1 | 1 | 0 | 1 | 0 | 1 | 0 | 1 | 0 | 7 |
| N. I. Gulyaev 2020 | 0 | 1 | 0 | 1 | 0 | 1 | 0 | 1 | 0 | 1 | 0 | 5 |
| Prapromporn Pinijmung 2022 | 1 | 1 | 0 | 1 | 0 | 1 | 0 | 1 | 0 | 1 | 0 | 6 |
| Wenxue Zhao 2020 | 1 | 1 | 1 | 0 | 0 | 1 | 0 | 0 | 0 | 1 | 0 | 5 |

AHRQ, Agency for Healthcare Research and Quality
