# Supplementary material for: Prevalence of sarcopenia and its association with clinical outcomes in heart failure: An updated meta‐analysis and systematic review
Source: Clin Cardiol. 2023 Jan 16;46(3):260–8. doi: 10.1002/clc.23970 (PMC10018088; doi:10.1002/clc.23970)
Supplement: Supplementary file 9 — Supplementary information. [file CLC-46-260-s011.pdf]

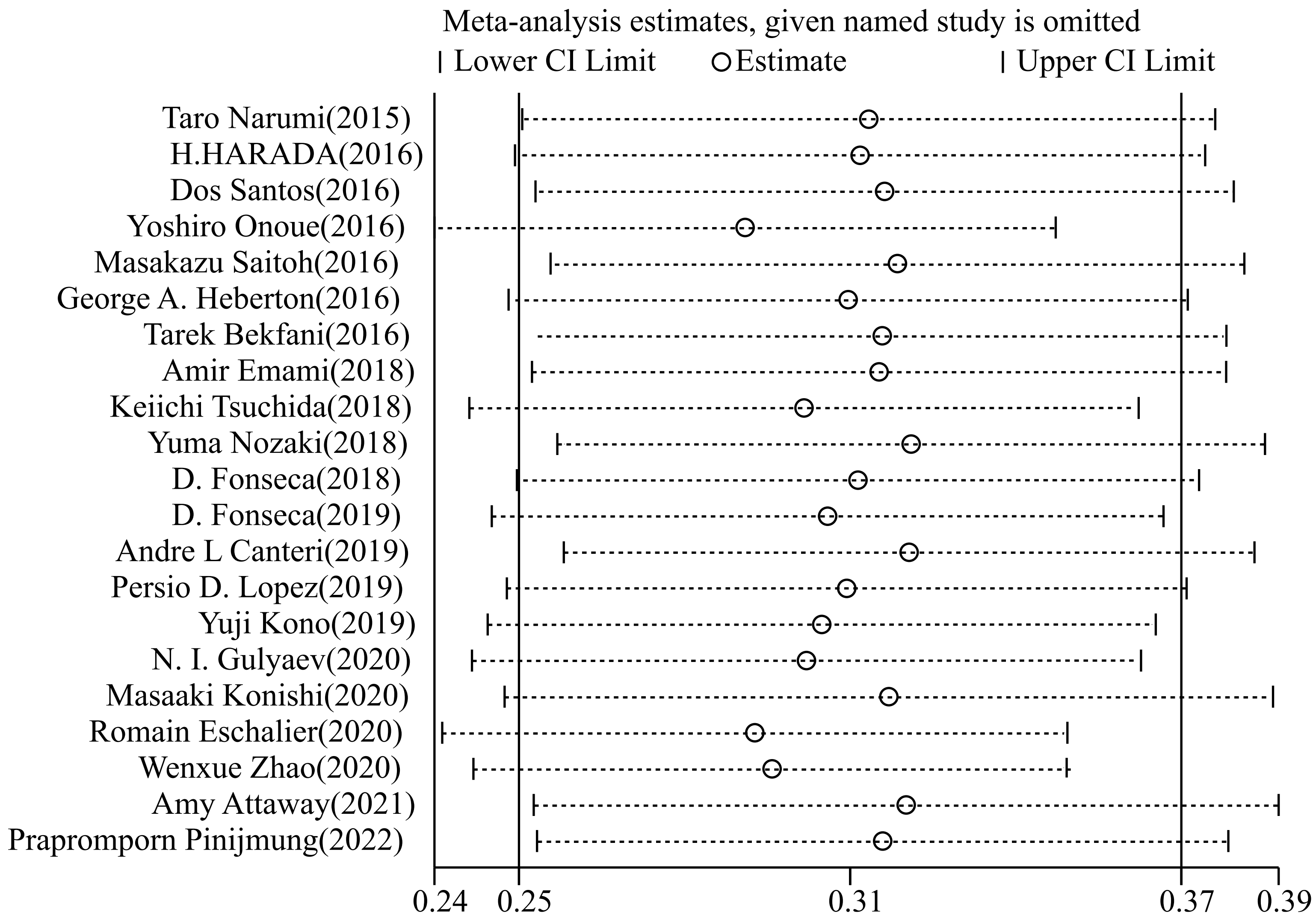

Supplementary Figure S9. Sensitivity analysis of prevalence of sarcopenia in heart failure.
